# Hepatocellular Carcinoma Cells Are Protected From Immunolysis by Mesenchymal Stromal Cells Through Indoleamine 2,3 Dioxygenase

**DOI:** 10.3389/fcell.2021.715905

**Published:** 2021-11-12

**Authors:** Raghavan Chinnadurai, Amanda Paige Porter, Mihir Patel, Ariel Joy Lipat, Mathews H. Forsberg, Devi Rajan, Peiman Hematti, Christian M. Capitini, Charles Bruker

**Affiliations:** ^1^Department of Biomedical Sciences, Mercer University School of Medicine, Savannah, GA, United States; ^2^Department of Pediatrics, Carbone Cancer Center, University of Wisconsin School of Medicine and Public Health, Madison, WI, United States; ^3^Department of Medicine, Carbone Cancer Center, University of Wisconsin School of Medicine and Public Health, Madison, WI, United States; ^4^Department of Pathology, Memorial Health University Medical Center, Savannah, GA, United States

**Keywords:** mesenchymal stromal cells, indoleamine 2,3 dioxygenase (IDO), hepatocellular carcinoma, B7 family checkpoints, immunotherapy

## Abstract

B7 family proteins serve as checkpoint molecules that protect tumors from T cell mediated lysis. Tryptophan degrading enzymes indoleamine 2,3 dioxygenase (IDO) and tryptophan 2,3 dioxygenase (TDO) also induce T cell immune tolerance. However, little is known about the relative contribution of B7 molecules, tryptophan degrading enzymes, as well as the impact of tumor and stromal cell interactions to the development of immunosuppressive tumor microenvironment. To investigate such interactions, we used a tripartite model of human hepatocellular carcinoma cell line (HepG2) and mesenchymal stromal cells (MSCs) co-cultured with peripheral blood mononuclear cells (PBMCs). Co-culture of HepG2 cells and activated PBMCs demonstrate that HepG2 cells undergo PBMC mediated cytolysis, despite constitutive expression of B7-H3 and upregulation of PD-L1 by IFNγ. Knockdown of B7-H3, PD-L1 or IDO does not modulate PBMC mediated lysis of HepG2 cells. However, TNFα preactivation enhances lysis of HepG2 cells, and blocking of TNFα production from PBMCs protects HepG2 cells. On the other hand, MSCs protect HepG2 cells from PBMC mediated lysis, even in the presence of TNFα. Further investigation showed that MSC mediated protection is associated with the unique secretome profile of upregulated and downregulated cytokines and chemokines. IFNγ activated MSCs are superior to TNFα activated or control MSCs in protecting HepG2 cells. Blockade of IFNγ driven IDO activity completely abolishes the ability of MSCs to protect HepG2 cells from cytolysis by PBMCs. These results suggest that inhibition of IFNγ activation of IDO induction in stromal cells, combined with usage of TNFα, could be a novel immunotherapeutic strategy to induce regression of hepatocellular carcinoma.

## Introduction

Hepatocellular carcinoma (HCC) is a cancer of the liver that is associated with significant mortality (Llovet et al., 2016). Although drugs such as sorafenib show promise in the treatment of HCC, patients with advanced stage disease still have very limited curative options (Faivre et al., 2020). Immunotherapeutic approaches are a promising alternative. Immune checkpoint blocking antibodies targeting the PD-1 and PD-L1 pathways show encouraging results for inducing HCC remissions and imply HCC tumors are immunogeneic (Greten and Sangro, 2017; Okusaka and Ikeda, 2018). However, targeting of PD-1 and PD-L1 pathway as monotherapy is not always responsive and combination therapy needs to be considered to overcome treatment resistance or non-responsiveness (Onuma et al., 2020; Merle, 2021). Hence, barriers other than PD-L1 and PD-1 within the tumor microenvironment that are preventing cytotoxicity by immune effector cells need to be further investigated.

Hepatocellular carcinoma primarily occurs as a consequence of chronic liver disease manifested by fibrosis and cirrhosis caused by hepatitis viral infections, chronic alcohol consumption, non-alcoholic fatty acid liver diseases, etc (Roth and Decaens, 2017; Ringelhan et al., 2018). Fibrosis is a process characterized by the accumulation of myofibroblasts/stromal cells in the liver which secrete collagen extracellular matrix, and lead to the hardening of tissue (Novikova et al., 2017). The origin of myofibroblasts/stromal cells in the liver is presumed to be largely from activated hepatic stellate cells, though bone marrow derived mesenchymal stem/stromal cells (MSCs) and fibrocytes also contribute to fibrogenesis. Russo et al. (2006) have shown that bone marrow functionally contributes to liver fibrosis. Another couple of studies have shown that MSCs can migrate to injured liver and can likely contribute to fibrogenesis (di Bonzo et al., 2008; Baertschiger et al., 2009). In addition, the contribution of bone marrow derived MSCs in promoting or inhibiting fibrogenesis was also discussed since MSCs are recruited to fibrotic tissue microenvironment (Kisseleva, 2017; Baglieri et al., 2019; Dhar et al., 2020). El Agha et al. (2017) suggest that MSCs may acquire epigenetic modifications upon chronic exposure to pro-inflammatory cytokines which render them profibrogenic potential in fibrosis development. Our earlier study had shown that MSCs and hepatic stellate cells share a majority of phenotypical and functional characteristics while some nuanced differences exist in their immune physiology (Chinnadurai et al., 2019). These studies suggest that MSCs could be a manipulator of hepatic fibrosis, cirrhosis and HCC.

Previous studies have demonstrated that bone marrow derived MSCs’ role in HCC pathogenesis is both stimulatory and inhibitory (Yin et al., 2018). MSCs were shown to inhibit HCC growth, proliferation, metastasis and induce apoptosis, and thus exhibit anti-HCC properties (Qiao et al., 2008; Zhao et al., 2012; Abdel Aziz et al., 2013; Yulyana et al., 2015). In contrary, MSCs were also shown to promote the proliferation, angiogenesis and epithelial to mesenchymal transition in HCC (Bhattacharya et al., 2012; Gong et al., 2013; Mi and Gong, 2017). Importantly, the interaction of MSCs with immune responders and the resulting effect on HCC fate is not well known. In the present study, we aim to test the hypothesis that MSCs confer dysfunctional anti-HCC T cell immunity which leads to the protection of HCC from immunolysis. Defining these will develop an immunotherapy strategy targeting stromal cells of HCC.

B7 family ligands provide costimulation and coinhibition to T cells and thus are the regulators of T cell immunity (Schildberg et al., 2016). There are many members present in the B7 family, namely, B7-1 (CD80), B7-2 (CD86), B7-H1 (PD-L1), B7-DC (PD-L2), B7-H2 (ICOSL), B7-H3, B7-H4 (B7x, B7S1), B7-H5, B7-H6, and B7-H7 which positively and negatively regulate T cell immunity (Sharma and Allison, 2015; Schildberg et al., 2016; Ni and Dong, 2017a,b). Our recent publication has demonstrated the correlation patterns of B7 family molecules in HCC microenvironment, which signifies the complex involvement of immunoregulatory molecules in modulating T cell immunity (Chinnadurai et al., 2020). Tryptophan degrading enzymes, indoleamine 2,3 dioxygenase (IDO) and tryptophan 2,3-dioxygenase (TDO), play a significant role in conferring T cell suppression and confer tumor immune tolerance (Munn and Mellor, 2016). Our recent publication has also demonstrated that IDO exhibits a strong correlative expression with PD-L1 in patients with HCC, which suggested that co-blocking of IDO and PD-L1 could be a promising strategy to promote anti-HCC T-cell immunity (Chinnadurai et al., 2020). However, the regulation and functionality of B7 family molecules and tryptophan degrading enzymes in modulating HCC fate in the context of MSCs are unknown. In the present study, we aim to define their significance in the tripartite interactions among liver cancer cells, MSCs and T cells.

## Materials and Methods

### Mesenchymal Stromal Cells and HepG2 Cells

Mesenchymal stromal cells were obtained from the bone marrow aspirates of healthy individuals according to institutional review board guidelines of Memorial Health University Medical Center and University of Wisconsin-Madison. Harvested bone marrow was separated by Ficoll density gradient and plated on α-MEM culture medium (Corning, NY, United States) containing 20% fetal calf serum (VWR, United States) and 100 U/ml penicillin/streptomycin (200,000 cell/cm^2^) (Corning, NY, United States). Non-adherent cells were removed from culture after 3 days and MSCs are allowed to expand for an additional 7 days. Subsequently, MSCs were passaged weekly and replated at a seeding density of 1,000 cells/cm^2^. All assays were performed using MSCs between passage 3 and 7. MSC identity was confirmed through flow cytometry as previously described (Chinnadurai et al., 2015; Supplementary Figure 1). HepG2 Cell line was obtained from American Type Culture Collection (MD, United States) and HepG2-Luciferase cell line (Bioware Brite Cell Line-HepG2-Red-FLuc) was obtained from PerkinElmer, United States (Waltham, MA, United States). All cell lines were mycoplasma tested every 5 months and deemed negative prior to use.

### Flow Cytometry

Bone marrow derived MSC cultures were assayed by flow cytometric analysis for the absence of CD45+ contaminating cells and expression of CD44, CD73, CD90, and CD105 (BD Biosciences, San Jose, CA, United States). Resting or IFNγ (20 ng/ml) (BioLegend, United States) or TNFα (20 ng/ml) (BioLegend, United States) activated MSCs or HepG2 cells (48–72 h) were subjected to flow cytometry analysis for the expression of B7-1 PE, B7-2 PE, B7-H1 PE, B7-DC PE, B7-H2 PE (BD Biosciences, San Jose, CA, United States), B7-H3 BV421, B7-H4 BV421, B7-H5 APC, B7-H6 APC, B7-H7 Alexa 647 and appropriate isotype controls were used for the background stainings (R&D systems, Minneapolis, MN, United States). Mean Fluorescent Intensity and histogram analysis for the marker expression was performed with Flow Jo software (BD Biosciences, San Jose, CA, United States).

### siRNA Knockdown on Human Mesenchymal Stromal Cells and HepG2 Cells

Mesenchymal stromal cells and HepG2-Luciferase cells were seeded in 96 well plates at a concentration of 10,000–20,000 cells per well 1 day prior to transfection with non-targeting control siRNA or B7-H1, B7-H3, IDO SMART Pool siRNA (Horizon Discovery, CO, United States). During transfection, the cells were conditioned with serum free 10 mM HEPES (Corning, NY, United States) containing α-MEM for 30 min. Two microliters of 100 μM specific/control siRNA solution(A) or 3 μl Dharmafect 1 reagent(B) (MSCs)/Dharmafect 4 reagent(B) (HepG2) was added in to 250 μl α-MEM containing 10 mM HEPES. A and B were mixed and incubated at room temperature for 30 min. 50 μl of the siRNA transfection cocktail was added to each well. The cells were then incubated for 5 h and the transfection medium was replaced with MSC/HepG2 culture medium. After 12 h, MSCs/HepG2 cells were stimulated with IFNγ. Subsequently 24 h later they were subjected to co-culture with Staphylococcal enterotoxin B (SEB) (Toxin Technology, FL, United States) activated PBMCs.

### Mesenchymal Stromal Cell, HepG2 and Peripheral Blood Mononuclear Cell Co-culture

Resting or IFNγ (20 ng/ml) or TNFα (20 ng/ml) activated MSCs or HepG2-Luciferase cells were seeded in 96 well plates at a concentration of 25,000–50,000 cells per well. Subsequently, 1000 ng/ml Staphylococcal enterotoxin B (SEB) (Toxin Technologies, United States) activated peripheral blood mononuclear cells (PBMCs) isolated from healthy individuals using an IRB approved protocol at the University of Wisconsin-Madison were added in different ratios. Two days later, 50 μl of 3 mg/ml D-Luciferin (Chem-Impex Inc., United States) was added into each well and the relative light units (RLU) were measured using the Veritas^TM^ Microplate luminometer (Turner BioSystems, United States). For co-culture with MSCs, 50,000 HepG2-Luciferase cells and 250,000 SEB activated PBMCs were added into a 24 well plate with appropriate numbers of resting or IFNγ (20 ng/ml) or TNFα (20 ng/ml) activated MSCs in the presence or absence of 1-methyl-DL-tryptophan (1 mM) (Sigma-Aldrich, United States). Two days later, total cells were subjected to the luciferase assay as described above.

### Statistical Analysis

Data were analyzed with the GraphPad Prism 8.0 software. Student t test was applied to compare the significance of mean values. P-value < 0.05 was considered statistically significant. Two-way analysis of variance (ANOVA) multiple-comparison test was performed between the independent conditions with the alpha of 0.05.

## Results

### Regulation of B7 Family Ligands and Tryptophan Degrading Enzymes in HepG2 Cells

**FIGURE 1:**
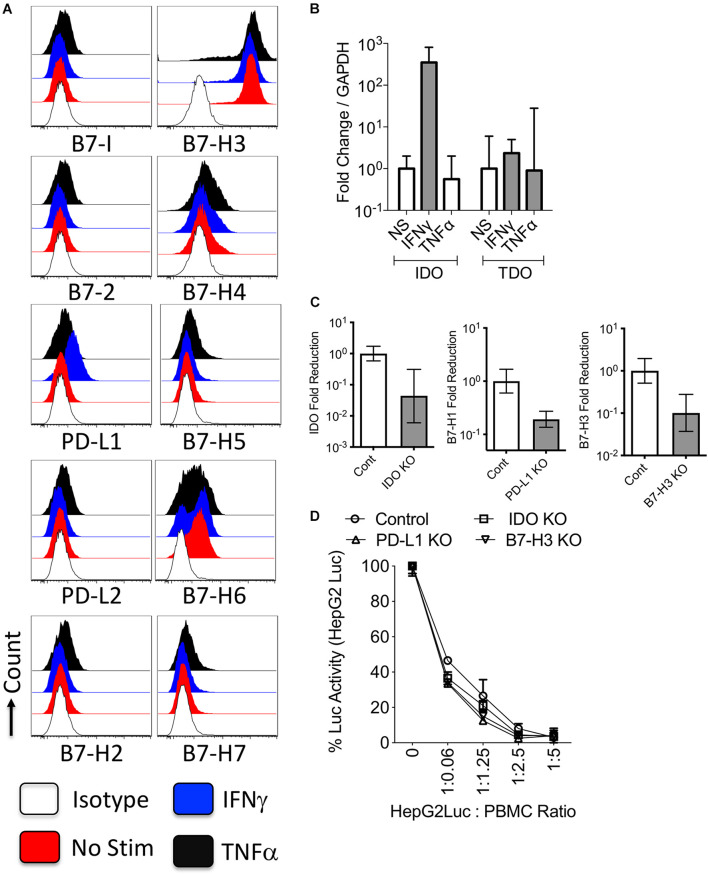
Regulation and functions of B7 family ligands and tryptophan degrading enzymes in the hepatocellular carcinoma cell line. HepG2 cell lines were treated with IFNγ or TNFα and surface expressions of B7 family ligands were analyzed through flow cytometry. **(A)** Histogram analysis shows the expressions of B7 family ligands in HepG2 cells in response to IFNγ and TNFα. Appropriate isotype controls are included. **(B)** HepG2 cells were stimulated IFNγ and TNFα for 48 h. The RNA was extracted from the cells and the expression levels of IDO and TDO mRNA was quantitated by the quantitative SYBR green real time PCR. GAPDH mRNA levels were used as internal controls. Delta-delta CT method was applied to calculate the fold change. **(C)** Control or IDO or PD-L1 or B7-H3 siRNA transfected HepG2 cells were stimulated with IFNγ for 48–72 h. Expression level of IDO, PD-L1, and B7-H3 mRNA relative to GAPDH was evaluated by quantitative SYBR green real time PCR. Delta-delta CT method was applied to calculate the fold reduction. **(D)** IDO or PD-L1 or B7-H3 or control siRNA transfected HepG2-Luciferase cells were cultured with SEB stimulated PBMCs. After 3 days, luciferase activity was measured. Mean and standard deviation are shown from technical replicates. Representative results from two independent experiments are shown.

To identify the constitutive and inducible expressions of B7 family molecules and enzymes of tryptophan degradation on HCC, we investigated their comparative expressions on HepG2 cells stimulated with cytokines IFNγ or TNFα which are commonly secreted by activated T cells. Flow cytometric analysis of resting HepG2 cells demonstrated that B7-H3 is constitutively expressed at high levels, while B7-H6 is expressed at low or moderate levels (Figure 1A and Supplementary Figure 2). IFNγ upregulated PD-L1 expression while TNFα had low/no effect. Other B7 family molecules were not modulated by IFNγ or TNFα (Figure 1A and Supplementary Figure 2). Next, we examined the expression of tryptophan degrading enzymes, IDO and TDO in HepG2 cells. Stimulation with IFNγ, but not TNFα, upregulated IDO expression on HepG2 cells (Figure 1B). However, TDO expression was not inducible with IFNγ or TNFα (Figure 1B).

### PD-L1, Indoleamine 2,3 Dioxygenase and B7-H3 Expression Do Not Protect HepG2 Cells From Peripheral Blood Mononuclear Cell Mediated Lysis

To decipher the role of IDO, PD-L1, and B7-H3 on HepG2 cells, we used siRNA knockdown approach (Figure 1C). We used HepG2-Red-FLuc cells which constantly express luciferase reporter enzyme that serves as the measure of their viability post co-culture with peripheral blood mononuclear cells (PBMCs). Control or target siRNA knockdown HepG2-Red-FLuc cells were co-cultured with PBMCs at various ratios. T cells in the PBMCs were activated with the super antigen Staphylococcus Enterotoxin B (SEB), which induces T cell proliferation and effector functions including the killing of target cells (HepG2-Red-FLuc cells). Relative Luciferase activity of HepG2-Red-FLuc cells co-cultured with/without activated PBMCs quantifies their viability or death mediated by activated PBMCs. Our results demonstrated that activated PBMCs dose dependently caused the immunolysis of control siRNA transfected HepG2-Red-FLuc cells (Figure 1D). Knockdown of IDO, PD-L1, and B7-H3 did not modulate the lysis susceptibility of HepG2-Red-FLuc cells to activated PBMCs (Figure 1D).

### TNFα Sensitization Enhances the Lysis of HepG2 Cells by Activated Peripheral Blood Mononuclear Cells

**FIGURE 2:**
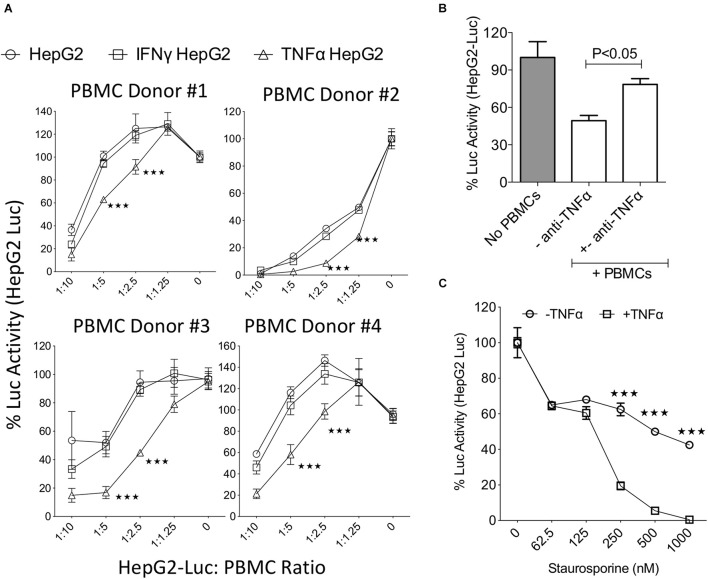
TNFα increases the susceptibility of HepG2 cells to lysis. Luciferase expressing HepG2 cells were treated with IFNγ and TNFα before co-culture with SEB activated PBMCs. Three days post co-culture luciferase activity was measured in a luminometer. **(A)** Dose dependent effect of SEB activated PBMCs from independent PBMC donors on the relative luciferase activity of IFNγ and TNFα treated HepG2-Luc cells were shown. Percentage luciferase activity was calculated based on the readouts of HepG2-Luc cells without PBMCs. Appropriate HepG2-Luc: PBMC ratio is shown in *X* axis and 0 indicates only HepG2-Luc without PBMCs. Two-way analysis of variance (ANOVA) multiple-comparison test was performed between the independent conditions. ***P ≤ 0.001. Mean and standard deviation are shown from technical replicates. **(A)** Represents cumulative data obtained from four independent PBMC donors. **(B)** Monoclonal blocking antibody to TNFα (50 μg/ml) was added in HepG2-Luc cultures with SEB activated PBMCs. Two days post culture, luciferase activity was measured. **(C)** ±TNFα activated HepG2-Luc cells were treated with the indicated concentrations of Staurosporine. Two days post culture, luciferase activity was measured. Two-way analysis of variance (ANOVA) multiple-comparison test was performed between the independent conditions. ***P ≤ 0.001. Mean and standard deviation are shown from technical replicates. Representative results were shown from two independent experiments.

To decipher the role of IFNγ and TNFα on the functional interaction of liver tumor cells with activated T cells, we utilized a co-culture system where IFNγ or TNFα activated HepG2-Red-FLuc were co-cultured with activated PBMCs. HepG2 cells displayed susceptibility to lysis by activated PBMCs in a dose dependent manner (Figure 2A). In addition, preactivation with IFNγ did not protect HepG2-Red-FLuc lysis by activated PBMCs (Figure 2A). However, preactivation with TNFα substantially augmented the lysis susceptibility of HepG2 cells to activated PBMCs (Figure 2A). Since activated PBMCs secrete large amount of TNFα, we hypothesized that TNFα produced by activated PBMCs increases the lysis susceptibility of HepG2 cells to activated PBMCs. To test this hypothesis, we performed TNFα neutralization in the HepG2-Red-FLuc and PBMC co-cultures. Blocking of TNFα with a monoclonal antibody increased the survival of HepG2-Red-FLuc cells co-cultured with activated PBMCs (Figure 2B). Next, we investigated if TNFα mediated sensitization of HepG2-Red-FLuc cells’ susceptibility to lysis also occurs in a non-immune dependent apoptosis mechanism. To test this, we primed HepG2-Red-FLuc cells with an apoptosis inducer, staurosporine, in the presence and absence of TNFα. Our results illustrate that co-incubation of staurosporine and TNFα showed an enhanced lysis than when used alone (Figure 2C). These results demonstrated that TNFα, but not IFNγ, increased the susceptibility of HepG2 cells to lysis through both immune and non-immune dependent mechanisms.

### Mesenchymal Stromal Cells Constitutively Express B7-H3 and IFNγ Upregulates B7-H1, B7-DC and Indoleamine 2,3 Dioxygenase

**FIGURE 3:**
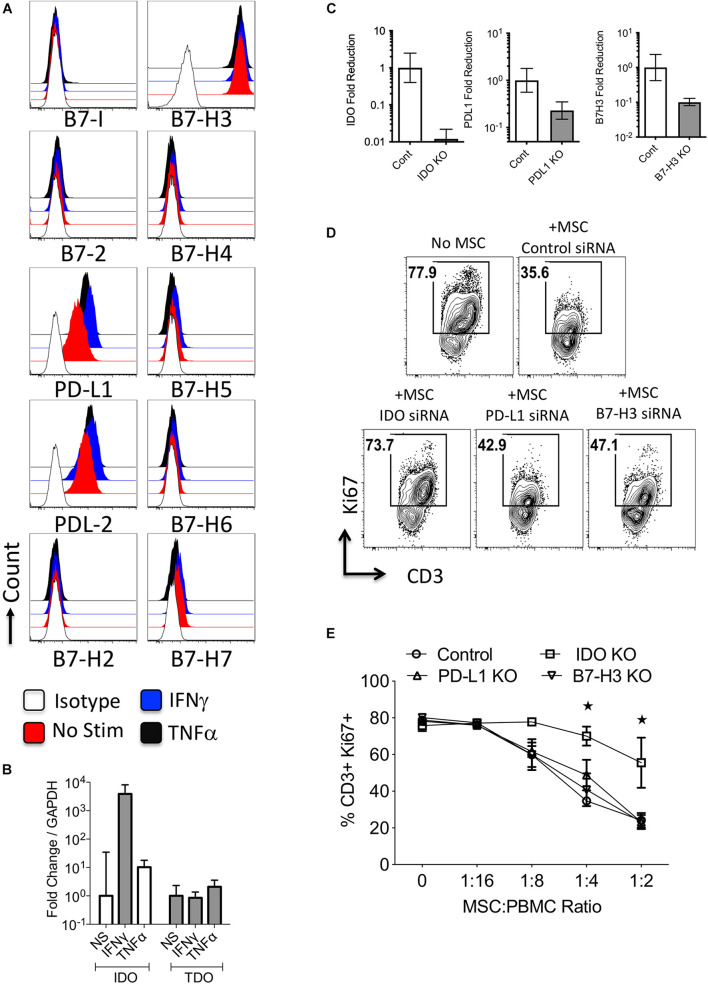
Regulation and functions of B7 family ligands and tryptophan degrading enzymes in Mesenchymal stromal cells. **(A)** Bone marrow derived MSCs were stimulated with IFNγ and TNFα and surface expressions of B7 family ligands were analyzed through flow cytometry. Histogram analysis shows the expressions of B7 family ligands on MSCs in response to IFNγ and TNFα. Appropriate isotype controls are included. **(B)** Bone Marrow derived MSCs were stimulated with IFNγ and TNFα for 48 h. The RNA was extracted from the cells and the expression levels of IDO and TDO mRNA was quantitated by the quantitative SYBR green real time PCR. GAPDH mRNA levels were used as internal controls. Delta-delta CT method was applied to calculate the fold change. **(C)** Control or IDO or PD-L1 or B7-H3 siRNA transfected MSCs were stimulated with IFNγ for 48–72 h. Expression level of IDO, PD-L1, and B7-H3 mRNA relative to GAPDH was evaluated by quantitative SYBR green real time PCR. Delta-delta CT method was applied to calculate the fold reduction. **(D)** IDO or PD-L1 or B7-H3 or control siRNA transfected MSCs were cultured with SEB stimulated PBMCs. After 4 days, T-cell proliferation was measured by Ki67 intracellular staining. **(D)** Representative FACS Plot and **(E)** dose dependent effect of MSCs on T cell proliferation is shown. Two-way analysis of variance (ANOVA) multiple-comparison test was performed between the independent conditions. *P ≤ 0.05. Mean and standard deviation are shown from technical replicates. Representative results are shown from two independent experiments.

To define the expression and regulation of B7 family members and tryptophan degrading enzymes in stromal cell components, we have investigated primary human bone marrow derived MSCs. Flow cytometry analysis has demonstrated that MSCs at resting stage express PD-L1 (B7-H1), PD-L2 (B7-DC), B7-H3, and low/moderate levels of B7-H7 (Figure 3A and Supplementary Figure 3). In addition, IFNγ substantially upregulated PD-L1 and PD-L2, while TNFα moderately upregulates these molecules. None of the other B7 family molecules were modulated by IFNγ or TNFα (Figure 3A and Supplementary Figure 3). Next, we have investigated the regulation of tryptophan degrading enzymes, IDO and TDO, in MSCs. IFNγ but not TNFα upregulated IDO while neither IFNγ nor TNFα modulate TDO (Figure 3B).

### Dominant Immunosuppressive Role of Indoleamine 2,3 Dioxygenase in Mesenchymal Stromal Cells Over PD-L1 and B7-H3

To further define the role of PD-L1, B7-H3, and IDO on MSCs immunosuppressive functions, we utilized a siRNA knockdown approach (Figure 3C). We co-cultured siRNA transfected MSCs with SEB activated PBMCs in varying ratios. Our results demonstrated that knocking down of IDO substantially reversed MSCs’ inhibitory effects on T-cell proliferation, while knocking down B7 family molecules on MSCs had minimal/no effect (Figures 3D,E). This demonstrates that IDO is necessary for MSC-mediated inhibition of T cell proliferation.

### Mesenchymal Stromal Cells Protect Control and TNFα Primed HepG2 Cells From Immunolysis

**FIGURE 4:**
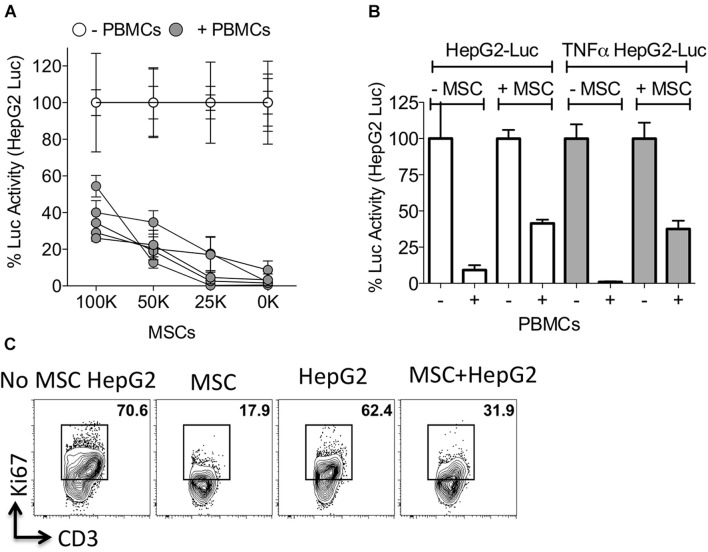
Mesenchymal stromal cells protect HepG2 cells from lysis. **(A)** HepG2-Luc cells and increasing doses of MSCs were cultured together with or without SEB activated PBMCs from independent donors. Three days later luciferase was measured to detect the survival of HepG2-Luc cells. Percentage luciferase activity was calculated based on the readouts of HepG2-Luc cells with appropriate doses of MSCs and without PBMCs. Mean and standard deviation are shown from technical replicates. **(A)** Represents cumulative data derived from five independent PBMC and MSC donors. **(B)** ±TNFα primed HepG2-Luc cells were co-cultured with or without MSCs and/or SEB activated PBMCs. Three days later luciferase was measured to detect the survival of HepG2-Luc cells. Mean and standard deviation are shown from technical replicates. Representative results are shown from three independent experiments. **(C)** MSCs and HepG2 cells were cultured together with SEB activated PBMCs. Four days later T-cell proliferation was measured by Ki67 intracellular staining and FACS plots are shown. Representative results are shown from two independent experiments.

To determine the effect of MSCs in protecting HepG2 cells from activated PBMC mediated lysis, we co-cultured HepG2-Red-FLuc cells with PBMCs in the presence of varying doses of MSCs. MSCs protected HepG2 cells from activated PBMC mediated lysis in a dose dependent manner (Figure 4A). Next, we investigated if MSCs also protect TNFα primed HepG2 cells from cytolysis by activated PBMCs. Our results show that MSCs inhibited the lysis of TNFα primed HepG2 cells upon co-culture with activated PBMCs (Figure 4B). To determine the direct effect of MSCs on T cell activity against HepG2, we co-cultured activated PBMCs with MSCs alone or with HepG2 cells. We observed an inhibitory effect on T-cell proliferation by MSC alone and in co-culture with HepG2 cells (Figure 4C). These results suggest that MSCs provide an immune-resistant tumor microenvironment by suppressing immune effector responses of PBMCs.

### Mesenchymal Stromal Cells Protect HepG2 Cells From Immunolysis With Secretome Modulation

Next, we aimed to define the secretome responses of MSC, PBMC, and HepG2 interaction. A secretome analysis of 30 cytokines and chemokines using xMAP^TM^ multiplex technology has identified that MSC and HepG2 co-culture modulated cytokines and chemokines upon interaction with activated PBMC cultures (Table 1). Upregulated and downregulated cytokines are hierarchically arranged based on the fold change between the cultures of activated PBMCs alone and co-culture with MSCs and HepG2. Basal secretome of MSCs and HepG2 in the absence of PBMCs are also shown in Table 1. TNFα, IL-5, IFNγ, MIP1α, and IL-2R are downregulated cytokines, while CXCL9, CXCL10, and GCSF are upregulated cytokines. These results suggested that MSC mediated protection of HepG2 cells from immune lysis confer unique modulation of secretome responses.

**TABLE 1:** Secretome of the tripartite interaction of MSCs, PBMCs, and HepG2 cells.

**Cytokines^#^**	**−PBMCs**				**+PBMCs**				Fold change*
	**HepG2**	**MSCs**	**MSCs + HepG2**	**No MSC No HepG2**	**HepG2**	**MSCs**	**MSCs + HepG2**	**No MSC No HepG2**	
TNFα	1 ± 0	2 ± 0	2 ± 0	1 ± 0	380 ± 11	19 ± 4	44 ± 7	584 ± 96	13.3^a^
IL-5	9 ± 0	1 ± 0	2 ± 0	9 ± 0	196 ± 37	13 ± 3	10 ± 2	92 ± 29	9.2^a^
IFNγ	4 ± 0	4 ± 0	0 ± 0	4 ± 0	3795 ± 0	350 ± 10	483 ± 5	2907 ± 73	6^a^
MIP1α	9 ± 0	21 ± 0	27 ± 5	12 ± 5	9740 ± 374	9215 ± 4305	10340 ± 1935	42638 ± 31938	4.1^a^
IL-2R	7 ± 4	39 ± 6	38 ± 8	20 ± 13	2124 ± 87	283 ± 114	210 ± 31	748 ± 113	3.6^a^
IL-17A	31 ± 0	31 ± 0	2 ± 1	31 ± 0	226 ± 26	27 ± 7	40 ± 10	109 ± 30	2.7^a^
EGF	7 ± 1	8 ± 0	7 ± 1	8 ± 0	17 ± 13	16 ± 4	10 ± 4	23 ± 10	2.3^a^
IL-13	14 ± 0	24 ± 0	24 ± 0	15 ± 1	184 ± 27	34 ± 2	35 ± 2	66 ± 7	1.9^a^
IL-10	3 ± 0	4 ± 0	5 ± 0	3 ± 0	711 ± 47	43 ± 1	93 ± 3	125 ± 34	1.3^a^
MIP-1β	17 ± 0	12 ± 0	21 ± 11	17 ± 0	24230 ± 5117	4892 ± 414	6094 ± 106	8172 ± 92	1.3^a^
IL-4	13 ± 1	23 ± 0	25 ± 2	13 ± 1	49 ± 4	31 ± 1	29 ± 1	38 ± 4	1.3^a^
GM-CSF	1 ± 0	3 ± 1	11 ± 1	1 ± 0	807 ± 77	374 ± 6	365 ± 39	473 ± 48	1.3^a^
IL-15	34 ± 1	29 ± 2	39 ± 4	34 ± 11	36 ± 26	61 ± 5	47 ± 1	60 ± 16	1.3^a^
IL-12/IL-23p40	10 ± 0	10 ± 0	5 ± 6	10 ± 0	498 ± 18	341 ± 66	286 ± 24	364 ± 19	1.3^a^
IFN-α	0 ± 0	4 ± 1	5 ± 0	2 ± 2	6 ± 1	5 ± 0	5 ± 0	4 ± 1	1.3^a^
IL-8	854 ± 34	2715 ± 42	2971 ± 90	11 ± 0	2748 ± 395	3092 ± 69	2915 ± 96	3107 ± 294	1.1^a^
CXCL9	5 ± 0	7 ± 3	7 ± 4	5 ± 0	1604 ± 18	33231 ± 2278	35518 ± 3926	98 ± 34	362.4^b^
CXCL10	4 ± 0	41 ± 39	12 ± 2	4 ± 0	4507 ± 6	4422 ± 243	4483 ± 38	48 ± 3	93.4^b^
G-CSF	51 ± 3	62 ± 3	64 ± 5	51 ± 3	1508 ± 6	4856 ± 226	4203 ± 532	71 ± 0	59.2^b^
VEGF	13 ± 0	426 ± 27	471 ± 8	3 ± 0	18 ± 0	486 ± 29	425 ± 51	8 ± 1	53.1^b^
FGF2	7 ± 0	7 ± 0	7 ± 0	7 ± 0	7 ± 0	419 ± 380	233 ± 186	7 ± 0	33.3^b^
HGF	7 ± 6	663 ± 62	957 ± 205	11 ± 0	37 ± 9	632 ± 135	524 ± 161	21 ± 5	25^b^
CCL5	28 ± 0	314 ± 120	98 ± 17	26 ± 5	1689 ± 297	3124 ± 103	2427 ± 5	1026 ± 18	2.4^b^
CCL2	20 ± 5	145958 ± 18429	132225 ± 17237	12 ± 7	72109 ± 7147	181020 ± 2802	184527 ± 1355	101171 ± 79732	1.8^b^
IL-1RA	175 ± 12	146 ± 3	157 ± 8	139 ± 3	1149 ± 30	640 ± 320	588 ± 158	328 ± 22	1.8^b^
IL-6	1 ± 1	13733 ± 571	14353 ± 441	10 ± 0	10237 ± 839	14862 ± 72	14390 ± 442	8411 ± 137	1.7^b^
IL-2	12 ± 0	12 ± 0	7 ± 8	12 ± 0	1019 ± 166	718 ± 23	921 ± 64	558 ± 80	1.7^b^
IL-1b	3 ± 0	4 ± 0	4 ± 0	3 ± 0	29 ± 2	37 ± 16	35 ± 8	25 ± 5	1.4^b^
IL-7	7 ± 2	15 ± 1	18 ± 1	5 ± 0	10 ± 2	16 ± 0	14 ± 2	10 ± 2	1.4^b^
CCL11	3 ± 0	8 ± 3	5 ± 0	3 ± 0	5 ± 1	6 ± 0	5 ± 1	4 ± 0	1.3^b^

*^#^Cytokine concentrations are given in pg/ml.*

*Fold change is calculated within the + PBMCs category between No MSC, No HepG2, and MSCs + HepG2 groups.

*^a^ Fold change in downregulation.*

*^b^ Fold change in upregulation.*

### Superiority of IFNγ Preactivated Mesenchymal Stromal Cells in Protecting HepG2 Cells

**FIGURE 5:**
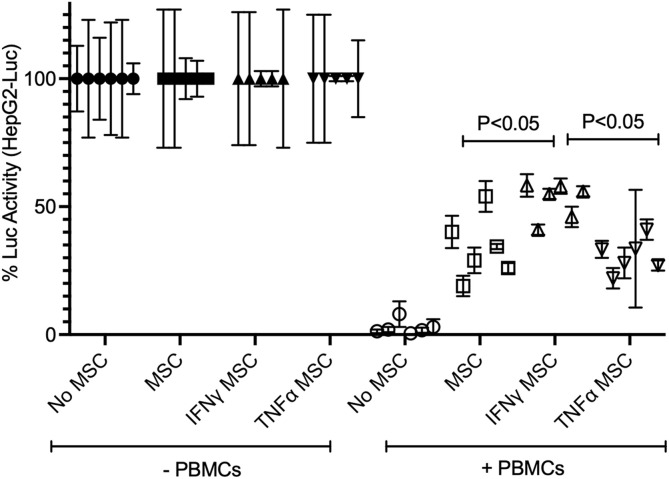
Superiority of IFNγ primed MSCs in protecting HepG2 cells from T cell mediated lysis. HepG2-Luc cells were co-cultured with ±IFNγ or ±TNFα treated MSCs with/without SEB activated PBMCs. Three days later luciferase was measured to detect the survival of HepG2-luciferase cells. Percentage luciferase activity was calculated based on the readouts of HepG2-Luc cells without PBMCs. Protective effects of ±IFNγ/TNFα MSCs on HepG2 cells are shown. Cumulative data with mean and standard deviation derived from six independent MSC donors are shown. Two tailed unpaired t test analysis was performed through prism software to get the *p* values.

Activated PBMCs produce high levels IFNγ and TNFα and modulate the expression of IDO and PD-L1 on MSCs (Table 1 and Figure 3). Hence, we aimed to determine the effect of IFNγ and TNFα on MSC-mediated HepG2’s resistance to PBMC mediated lysis. We compared resting (control), IFNγ and TNFα preactivated MSCs in inhibiting SEB activated PBMC mediated lysis of HepG2-Red-FLuc cells. Resting, IFNγ, and TNFα treated MSCs protected HepG2 cells from activated PBMC mediated lysis. In addition, IFNγ MSCs showed a superior protective effect compared to the other MSC groups (Figure 5). These results suggested the superiority of IFNγ preactivated MSCs in protecting HepG2 cells from immunolysis.

### Mesenchymal Stromal Cells Protect HepG2 Cells Predominantly Through Indoleamine 2,3 Dioxygenase

**FIGURE 6:**
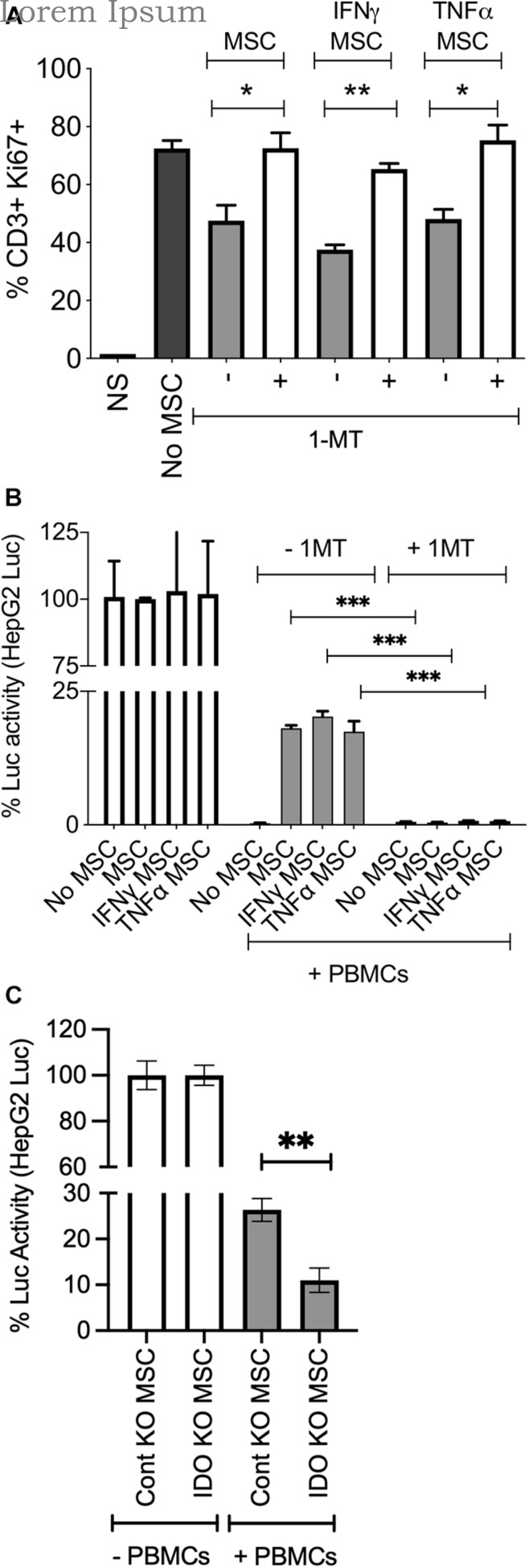
MSCs protect HepG2 cells through indoleamine 2,3 dioxygenase mechanism. ±IFNγ or±TNFα treated MSCs were cultured with HepG2-Luc cells and SEB activated PBMCs with and without 1-MT (1 mM). After 3 days, **(A)** T cell proliferation was measured by Ki67 intracellular staining. Two tailed unpaired t test analysis was performed through prism software to get the *p* values. **(B)** Luciferase activity was measured to detect the survival of HepG2 cells on the third day. Two-way analysis of variance (ANOVA) multiple-comparison test was performed between the independent conditions using Prism Software. **(C)** Control or IDO siRNA transfected MSCs were cultured with HepG2-Luc cells in the presence and absence of SEB stimulated PBMCs. Luciferase activity was measured to detect the survival of HepG2 cells on the third day. Two tailed unpaired t test analysis was performed through prism software to get the *p* values. *P ≤ 0.05, **P ≤ 0.01, ***P ≤ 0.001. Representative results are shown from two independent experiments.

IFNγ substantially upregulates IDO on MSCs (Figure 3). In order to determine if IDO produced by MSCs protect HepG2 cells from activated T cell mediated lysis, we blocked IDO activity with the pharmacological inhibitor 1-methyl tryptophan (1-MT) as previously described (Chinnadurai et al., 2016). Blocking of IDO activity completely abolished the protective effect of resting, IFNγ and TNFα primed MSCs on HepG2 cells, and restored T cells proliferation (Figures 6A,B). To further define the specific role of IDO on MSCs in protecting HepG2 cells from immunolysis, we utilized a siRNA knockdown approach. We co-cultured control or IDO siRNA transfected MSCs with HepG2-Red-FLuc cells in the presence and absence of SEB activated PBMCs. Our results have demonstrated that IDO siRNA transfected MSCs fail to protect HepG2 cells from immunolysis mediated by PBMCs (Figure 6C). These results demonstrated that IDO produced by stromal cells directly inhibits T cell functionality and facilitates an immunosuppressive microenvironment within HCC.

## Discussion

Immunotherapy to revive dysfunctional/exhausted anti-tumor immunity is a promising strategy for many different types of cancers including HCC (Singh et al., 2021). U.S. Food and Drug Administration (FDA) and European Medicines Agency (EMA) have approved immunotherapeutic agents targeting the PD-L1, PD-L2/PD1 pathway in HCC patients (Sangro et al., 2021). Although the results are encouraging, treatment resistance and non-response is a concern (Dong et al., 2020). One of the potential reasons for these discordances could be due to the involvement of other dominant immunoregulatory molecules that play along with PD-L1/PD1 pathway in HCC microenvironment (Lafleur et al., 2018; Heinrich et al., 2020; Sharma and Motedayen Aval, 2021). Our recent work has identified that PD-L1 is the only molecule in B7 family which exhibits a high correlative expression with tryptophan degrading enzyme, IDO in HCC (Chinnadurai et al., 2020). These findings suggest that IDO could play a role in protecting HCC cells from anti-tumor T cell immunity, which could be an explanation for the inconsistent efficacy of anti-PDL1/anti-PD-1 monotherapy. The present study provided evidence that IDO in stromal cells protects HCC from immunolysis.

The liver tumor microenvironment is comprised of stromal cells, tumor cells and infiltrating immune effector cells such as T cells. In this intricate immunosuppressive microenvironment dysfunctional anti-HCC T cell immunity leads to the failure of the immune system to eliminate HCC tumor cells. The involvement of stromal component of solid tumors in protecting tumor cells from anti-tumor immune responses is increasingly being appreciated (Shi et al., 2017). Thus, stroma of the HCC microenvironment could be an attractive target for immunotherapeutic approach (Heindryckx and Gerwins, 2015). Stromal cells play an important role in the disease progression of chronic liver damage, manifested by fibrosis and cirrhosis which leads to the development of HCC (Yin et al., 2018; Ganne-Carrie and Nahon, 2019). To determine the role of B7 family molecules and tryptophan degrading enzymes (IDO, TDO) in tumor and stromal cell interactions, we investigated the response of HepG2 cells and MSCs to cytokines commonly produced by infiltrating immune effector cells, such as IFNγ and TNFα. Both of these cytokines have no effect on the levels of costimulatory molecules B7-1 and B7-2 on HepG2 and MSCs. However, IFNγ upregulates PD-L1 and IDO on both cell types and PD-L2 is upregulated on MSCs. These results suggest that liver cancer cells and stromal cells provide immunosuppressive functions not only through the lack of costimulatory molecule expression, but also due to the abundant expression of IDO, PD-L1 and PD-L2, which collectively suppress inflammation.

Our functional analyses demonstrate that HepG2 cells are susceptible to cytolysis by activated PBMCs, and that this lysis is further enhanced by TNFα. This increased sensitivity to TNFα is also observed with the apoptotic inducer, staurosporine. Previous studies have shown that TNFα synergistically enhances the apoptotic susceptibility of HCC cells to commonly used anti-cancer drugs like Taxol (Paclitaxel) (Minero et al., 2015). In addition, over expression of TNFα induced A20 in HCC cells enhances their radiation sensitivity (Liu et al., 2017). Our multiplex secretome analysis confirmed that the protection of HepG2 cells from activated PBMC mediated lysis is associated with the downregulation of TNFα. All these results support the hypothesis that TNFα enhances the susceptibility of HCC cells to immune mediated lysis and TNFα is a potent anti-HCC cytokine.

Mesenchymal stromal cells migrate to the sites of inflammation, enhance malignant proliferation, and contribute to the expansion of the fibrovascular network and tumor progression (Spaeth et al., 2009; Jafari et al., 2021). It is also known that MSCs protect cancer cells through TGF-beta dependent regulatory T cell mediated immune suppression (Patel et al., 2010). Here we demonstrated that MSCs protect HCC cells from activated PBMC mediated immunolysis even in the presence of immunolysis enhancer TNFα. In addition, IFNγ primed MSCs do protect HCC cells from PBMC mediated lysis more substantially than the other resting or TNFα primed MSCs. IFNγ upregulates IDO on MSCs, and blocking of IDO completely abolishes the protective effect of MSCs on HCC cells, suggesting that IDO plays an important role in the stromal cell mediated immune suppression seen in the tumor microenvironment.

We also observed that HepG2 cells in the absence of MSCs are susceptible to immunolysis by activated PBMCs despite the constitutive expression of B7-H3 and upregulation of IDO and PD-L1 by IFNγ. In addition, knockdown of PD-L1, B7-H3 and IDO on HepG2 cells does not increase their susceptibility to activated PBMC mediated lysis. However, blocking of IDO activity in MSCs completely abolishes MSC’s protective effect on HepG2 cells from immunolysis. IDO’s role in tumor cells, macrophages, and dendritic cells has been well demonstrated in anti-tumor immunity (Munn and Mellor, 2004, 2007; Mellor et al., 2017). Our results show the relative significance of IDO produced by MSCs and its contribution to the protection of HCC cells from immunolysis. Although the present study utilized the racemic mixture of 1-MT (1-Methyl-DL-Tryptophan) as a pan inhibitor of IDO, future studies are warranted to identify the relative effects of D and L enantiomers and preclinical pipeline IDO inhibitors (Le Naour et al., 2020) in promoting HCC immunolysis.

Our results also show that IFNγ and TNFα have opposite effects in the tumor microenvironment. TNFα promotes the cytolysis of tumor cells while IFNγ upregulates immunosuppressive molecules to dampen the anti-tumor immune response. Although TNFα sensitizes HepG2 cells to lysis by activated PBMCs, the presence of MSCs abrogates cytolysis of TNFα sensitized HepG2 cells. In addition, IFNγ preactivated MSCs provide superior protection of HepG2 cells compared to resting and TNFα sensitized HepG2 cells. Sole blocking of IFNγ induced IDO activity completely abolishes MSC’s protection on HepG2 cells from immune lysis. Thus, our results suggest that immunotherapeutic approaches should aim to augment TNFα rather than IFNγ in the tumor microenvironment, and that MSCs may be a barrier to stimulating effector cells within the tumor microenvironment.

One of the limitations of our study is the lack of evidence on the role of stromal cells in protecting tumor cells in *in vivo* animal model studies. Our previous studies have shown that there are dissimilarities between murine and human stromal cells of marrow and liver in executing immunosuppressive pathways. Human MSCs inhibit T cell proliferation through IDO but not through inducible nitric oxide synthase (iNOS). However, murine derived counterparts display differences since murine MSCs inhibit T cell proliferation through iNOS but not IDO (Chinnadurai et al., 2019). These suggest that careful deliberations are required for the clinical translation of *in vivo* animal studies of stromal cells into the human immune physiology. Another limitation of our study is the lack of demonstration of immune lysis effect by HCC antigen specific T cell responses. However, technically these experiments are challenging since HCC tumor antigen specific T cells are low in frequency and also display dysfunctional and exhausted phenotype. Utilization of exhausted/dysfunctional tumor antigen specific T cells in the present assay system does not define the role of stromal cells and immunoregulatory molecules in protecting HCC. In addition, future work is also warranted to define the relative role of cancer associated fibroblasts, myofibroblasts in modulating the susceptibility of HepG2 and other HCC cell lines to immunolysis. Nevertheless, with the tripartite MSC, HepG2, and PBMC activation system, we were able to the present the role of MSCs in protecting HCC cells. In conclusion, our study provides evidence for targeting IDO within stromal cells during immunotherapy of HCC.

## Data Availability Statement

The original contributions presented in the study are included in the article/Supplementary Material, further inquiries can be directed to the corresponding author.

## Ethics Statement

The studies involving human participants were reviewed and approved by the University of Wisconsin-Madison. Written informed consent for participation was not required for this study in accordance with the national legislation and the institutional requirements.

## Author Contributions

RC conceived and designed the studies, performed the experiments, analyzed and interpreted the data, and drafted the manuscript. AP, MP, and AL performed the experiments related to MSCs. DR helped with Luminex Analysis. MF and CC helped with human PBMC preparations. PH and CB provided bone marrow aspirates. All authors contributed to editing of the manuscript.

## Conflict of Interest

CC receives honorarium from Nektar Therapeutics and Novartis for advisory board membership. The remaining authors declare that the research was conducted in the absence of any commercial or financial relationships that could be construed as a potential conflict of interest.

## Publisher’s Note

All claims expressed in this article are solely those of the authors and do not necessarily represent those of their affiliated organizations, or those of the publisher, the editors and the reviewers. Any product that may be evaluated in this article, or claim that may be made by its manufacturer, is not guaranteed or endorsed by the publisher.
