# Supplementary material for: Hepatocellular Carcinoma Cells Are Protected From Immunolysis by Mesenchymal Stromal Cells Through Indoleamine 2,3 Dioxygenase
Source: Front Cell Dev Biol. 2021 Nov 12;9:715905. doi: 10.3389/fcell.2021.715905 (PMC8633446; doi:10.3389/fcell.2021.715905)
Supplement: Supplementary file 1 [file Image_1.pdf]

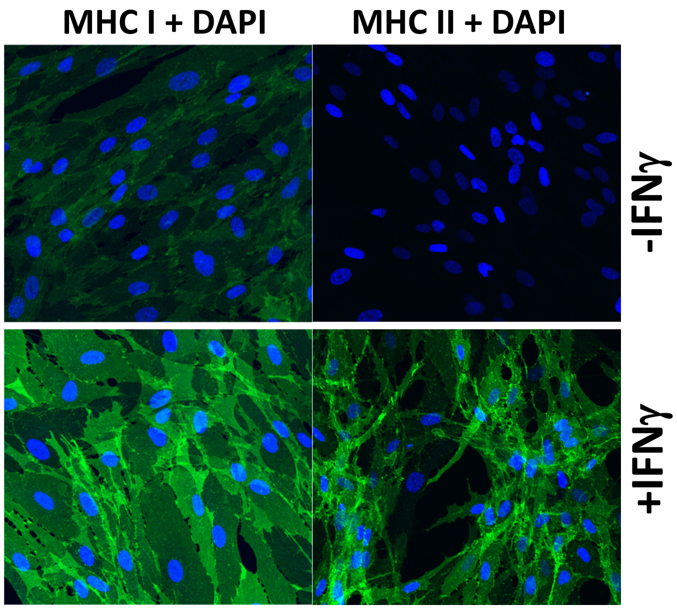


Figure S1. Microscopic analysis of MHC class I, MHC Class II (HLADR) expression on MSCs stimulated with and without IFNγ. 20 X magnification. Green: MHC Class 1 or MHC class II, Blue: DAPI.
