# Supplementary material for: Hepatocellular Carcinoma Cells Are Protected From Immunolysis by Mesenchymal Stromal Cells Through Indoleamine 2,3 Dioxygenase
Source: Front Cell Dev Biol. 2021 Nov 12;9:715905. doi: 10.3389/fcell.2021.715905 (PMC8633446; doi:10.3389/fcell.2021.715905)
Supplement: Supplementary file 3 [file Image_3.pdf]

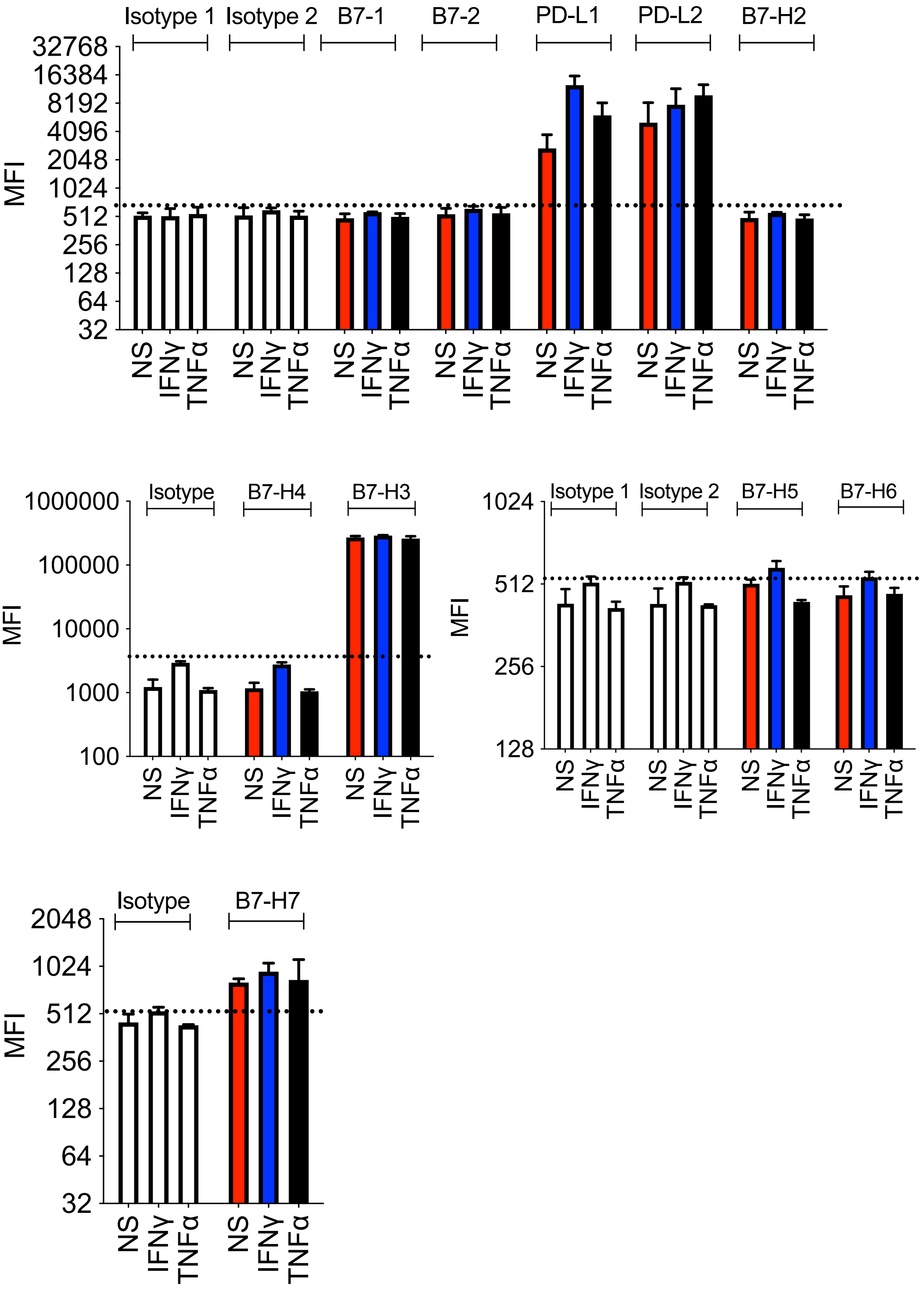

Figure S3. B7 family ligand expression on MSCs stimulated with IFNγ or TNFα is shown with Mean Fluorescence Intensity (MFI) and standard deviation. n= Two independent MSC donors. MFI of isotype controls for the respective B7 family molecules are also shown. Dotted lines represent background MFI with reference to isotype control staining. Please also refer main figure 3A for histograms.
